# An Antidote to Cocaine

**Published:** 1894-12

**Authors:** J. G. Gundlach

**Affiliations:** Spokane, Washington


### AN ANTIDOTE TO COCAINE.

J. G. Gundlach, M. D., Spokane, Washington.

I was called to attend a young woman who was suffering from the effects of Cocaine, administered by her dentist, with the following symptoms: Shortly after giving a small injection of a ten per cent. solution of Cocaine into her gum the doctor noticed that she at once passed off into a stupor, from which he could not arouse her, accompanied with some difficulty in her breathing and somewhat feeble heart action. The doctor gave some Spirits of Ammonia and fanned her, but she kept getting worse. At this time I was called in to assist him. Now spasmodic symptoms began to manifest themselves in the hands and arms, which began to tremble and soon to jerk. The breathing became more difficult and the spasm would reach the larnyx, when it (the breathing) would stop, and she would make great effort to get her breath by throwing her head back and grasping at her neck with her hands, her mouth wide open. The spasm, which lasted so long that we feared she would suffocate, would pass off with several short, loud, crowing inspirations which would end the attack, stupor with inability to swallow remaining during the interval. After a minute or two the above conditions would be repeated. I first gave whisky, but watching her symptoms, the symptoms of Gelsemium: of *stupor, inability to swallow and spasm of the larynx*—came to my mind, which I gave her, and stopped all other treatment. It soon began to do its work; only one or two hard spells after giving her Gelsemium, when they became lighter and farther apart, consciousness returned and with it the power to swallow and to speak. After about an hour's work we sent her home. She with the assistance of a friend walked down two flights of stairs to a carriage.

I have seen several other cases under the effects of Cocaine, but none manifested the stupor, inability to speak and swallow, with spasm of the larynx, all symptoms *characteristic of Gelse-*

*mium.* I believe I have discovered the much-needed antidote to Cocaine. So much for "*Similia similibus curantur.*" Any one wishing to know what preparation I gave can find out by writing me, enclosing stamp.

